# Gain-of-function mutations in KATP channel subunits compromise colonic tight junction integrity and epithelial homeostasis in murine models of Cantú syndrome

**DOI:** 10.3389/fmed.2025.1656718

**Published:** 2025-09-11

**Authors:** Fatima Maqoud, Domenica Mallardi, Antonella Orlando, Domenico Tricarico, Colin G. Nichols, Marina Antonacci, Giusy Bianco, Raffaele Armentano, Ilaria Grassi, Anna Maria Valentini, Francesco Russo

**Affiliations:** ^1^Functional Gastrointestinal Disorders Research Group, National Institute of Gastroenterology IRCCS “Saverio de Bellis”, Castellana Grotte, Bari, Italy; ^2^Section of Pharmacology, Department of Pharmacy-Pharmaceutical Sciences, University of Bari Aldo Moro, Bari, Italy; ^3^Department of Cell Biology and Physiology, and Center for the Investigation of Membrane Excitability Diseases (CIMED), Washington University, St. Louis, MO, United States; ^4^Animal Facility, National Institute of Gastroenterology, IRCCS “Saverio de Bellis” Research Hospital, Castellana Grotte, Bari, Italy; ^5^Histopathology Unit, National Institute of Gastroenterology IRCCS “Saverio de Bellis”, Castellana Grotte, Bari, Italy

**Keywords:** Cantú syndrome, murine model, ion channels, KATP channels, gain-of-function mutation, tight junctions, colonic epithelium, barrier dysfunction

## Abstract

**Introduction:**

Cantú syndrome (CS) is a rare genetic disorder caused by gain-of-function (GOF) mutations in the *KCNJ8* (Kir6.1) or *ABCC9* (SUR2) subunits of ATP-sensitive potassium (KATP) channels. CS is characterized by multisystem abnormalities such as cardiovascular defects, hypertrichosis, and skeletal malformations, but its impact on intestinal homeostasis remains poorly understood.

**Methods:**

We investigated the effects of CS-associated KATP channel overactivity on epithelial barrier integrity and tight junction (TJ) proteins using murine models. Heterozygous (SUR2^wt/AV^) and homozygous (SUR2^AV/AV^) SUR2(A478V) mutants, as well as Kir6.1(V65M) mice, were studied. mRNA and protein expression of Occludin, Claudin-1, and ZO-1 were analyzed, alongside histological and immunohistochemical assessments. Markers of apoptosis and survival, including caspase-3 activity and BCL2/BCL2L1 expression, were also evaluated.

**Results:**

GOF mutations in KATP channels caused significant dysregulation of TJ proteins. Occludin expression was increased in SUR2^AV/AV^ mice but decreased in SUR2^wt/AV^ and Kir6.1 mutants, while Claudin-1 and ZO-1 were consistently reduced across all models. Immunohistochemistry revealed disrupted TJ localization and reduced apical junctional integrity. Histological analyzes showed epithelial disorganization, smooth muscle hypertrophy, fibrosis, and inflammatory infiltration. These alterations were accompanied by increased caspase-3 activity and reduced BCL2 and BCL2L1 expression.

**Discussion:**

Our findings demonstrate that CS-associated KATP channel GOF mutations disrupt tight junction dynamics and induces structural remodeling of the colon. This establishes a novel link between KATP channel dysregulation, metabolic-epithelial interactions, and intestinal pathophysiology in CS. Furthermore, the results highlight potential therapeutic targets to mitigate barrier dysfunction, providing a basis for developing interventions to address gastrointestinal symptoms in CS.

## Introduction

Cantú syndrome (CS, OMIM 239850), first described in 1982, is a rare multisystem disorder caused by gain-of-function (GOF) mutations in subunits of ATP-sensitive potassium (KATP) channels, specifically the pore-forming Kir6.1 (KCNJ8) and regulatory SUR2 (ABCC9) subunits (1–4). Clinically, CS is characterized by distinctive craniofacial dysmorphisms, cardiovascular anomalies, hypertrichosis, and skeletal malformations (5–7). Electrophysiological analyses have demonstrated that disease-associated mutations enhance channel activity via reduced MgATP-mediated inhibition and heightened MgADP sensitivity, leading to aberrant cellular excitability (2, 8–10). While no targeted treatments are currently available, diagnosis is facilitated by clinical evaluation and genetic testing. Prognosis is variable and largely determined by cardiovascular involvement, although many individuals experience favorable outcomes with appropriate care.

KATP channels function as metabolic sensors, linking intracellular nucleotide concentrations to membrane excitability. These octameric complexes consist of four inwardly rectifying Kir6.x subunits (Kir6.1/KCNJ8 or Kir6.2/KCNJ11) and four sulfonylurea receptor subunits (SUR1/ABCC8 or SUR2/ABCC9). Their tissue-specific configurations confer functional specialization across the pancreatic, cardiovascular, skeletal, and neural systems (11–14). Clinically, KATP channels are important pharmacological targets, with sulfonylureas and potassium channel openers used to treat conditions such as diabetes, angina, and hypertension (10, 15–17).

In the gastrointestinal tract, KATP channels regulate chloride secretion, influence smooth muscle contractility, and modulate neural reflexes, playing a role in disorders like inflammatory bowel disease and Hirschsprung's disease (18–23). Strong expression of KATP subunits has been observed in the human colon. Specifically, Kir6.2/SUR1 is predominantly localized to the colonic epithelium, modulating fluid transport through ATP-sensitive chloride secretion (20, 24, 25). In contrast, Kir6.1/SUR2B is enriched in colonic smooth muscle, influencing contractile tone in response to ATP/ADP dynamics (26–28). Within the enteric nervous system, Kir6.2/SUR1 is expressed in inhibitory neurons of the myenteric and submucosal plexuses, dampening excitability and modulating reflex pathways (29–31). Meanwhile, Kir6.1/SUR2B subunits localize to interstitial cells of Cajal (ICC), where they may support pacemaker activity and coordinate motility (32–36).

Rodent studies support a role for colonic KATP channels in chloride secretion and epithelial barrier maintenance, with functional interactions noted between these channels and hydrogen sulfide (H_2_S) signaling (37). Co-localization of Kir6.1/SUR2 with tight junction (TJ) proteins in the small intestine further suggests involvement in barrier integrity (20, 38). Tight junctions comprising transmembrane proteins such as claudins and occludin, and scaffold proteins including ZO-1, ZO-2, and ZO-3, regulate epithelial permeability and are frequently disrupted in intestinal inflammation (39–42). These observations indicate a potential role for KATP channel dysregulation in epithelial pathophysiology.

Our previous work demonstrated that CS-associated GOF mutations in murine KCNJ8 [Kir6.1(V65M)] and ABCC9 [SUR2(A478V)] recapitulate key human phenotypes, including profound skeletal muscle pathology and cardiovascular defects (43, 44). However, the impact of these mutations on intestinal homeostasis, particularly within the colonic epithelium and junctional architecture, remains insufficiently understood.

The Kir6.1(V65M) and SUR2(A478V) mutations likely perturb colonic physiology via distinct mechanisms. Kir6.1(V65M) may enhance channel open probability, promoting excessive potassium efflux and membrane hyperpolarization, impairing ion transport and TJ integrity. In contrast, SUR2(A478V) may decrease ATP sensitivity, resulting in constitutive channel activation, metabolic dysregulation, oxidative stress, and caspase-3-dependent apoptosis. These divergent mechanisms suggest mutation-specific effects on epithelial and barrier function. To investigate this, we examined murine models harboring heterozygous (SUR2^wt/AV^) and homozygous (SUR2^AV/AV^) SUR2(A478V) alleles, as well as Kir6.1(V65M) variants. Our study aimed to define the impact of aberrant KATP channel activity on the assembly, localization, and function of tight junction components within the colonic epithelium.

## Results

### Kir6.1- and SUR2-dependent KATP channels are expressed at the epithelial level and the muscular level in the colon

**Figure 1:**
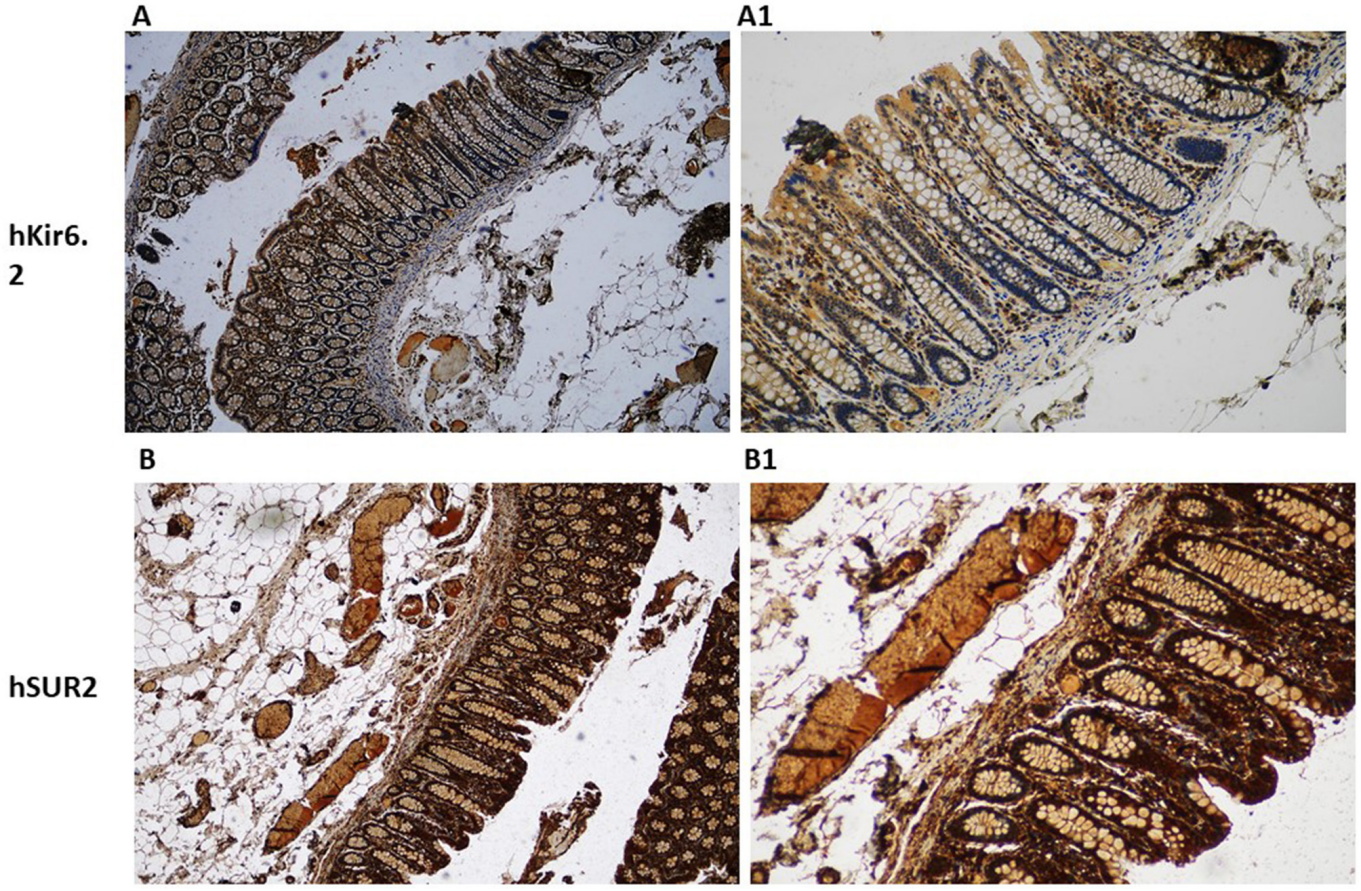
Immunohistochemical localization of KATP channel subunits Kir6.1 and SUR2 in normal human colon tissue. Representative immunohistochemical staining showing the expression of the KATP channel subunits Kir6.1 **(A, A1)** and SUR2 **(B, B1)** in histological sections of normal human colon. Images were acquired at low magnification [10×; **(A, B)**] to provide an overview of tissue architecture and at higher magnification [20×; **(A1, B1)**] to highlight cellular localization.

**Figure 2:**
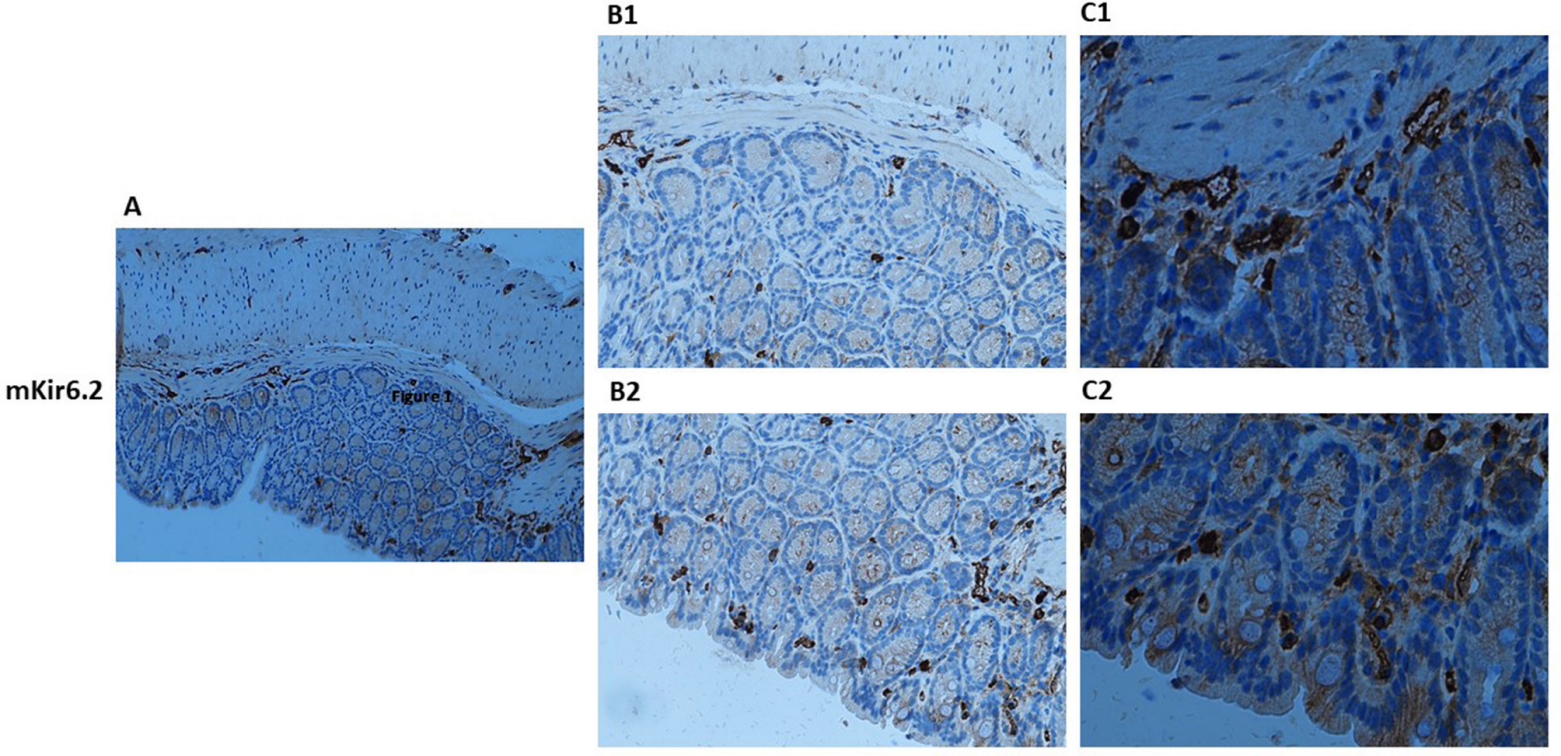
Immunohistochemical localization of KATP channel Kir6.1 subunit in normal mouse colon tissue. Representative immunohistochemical staining illustrating the distribution of KATP channel Kir6.1 subunit in histological sections of normal mouse colon at varying magnifications. **(A)** shows low-magnification imaging (10×) to provide an overview of overall tissue morphology. **(B1, B2)** (20×) and **(C1, C2)** (40×) present higher magnification views to resolve specific regional expression. At 20× and 40× magnification, Kir6.1 immunoreactivity is observed in distinct layers of the colon, including the lamina propria and muscularis **(B1, C1)**, as well as within the crypt structures **(B2, C2)**.

**Figure 3:**
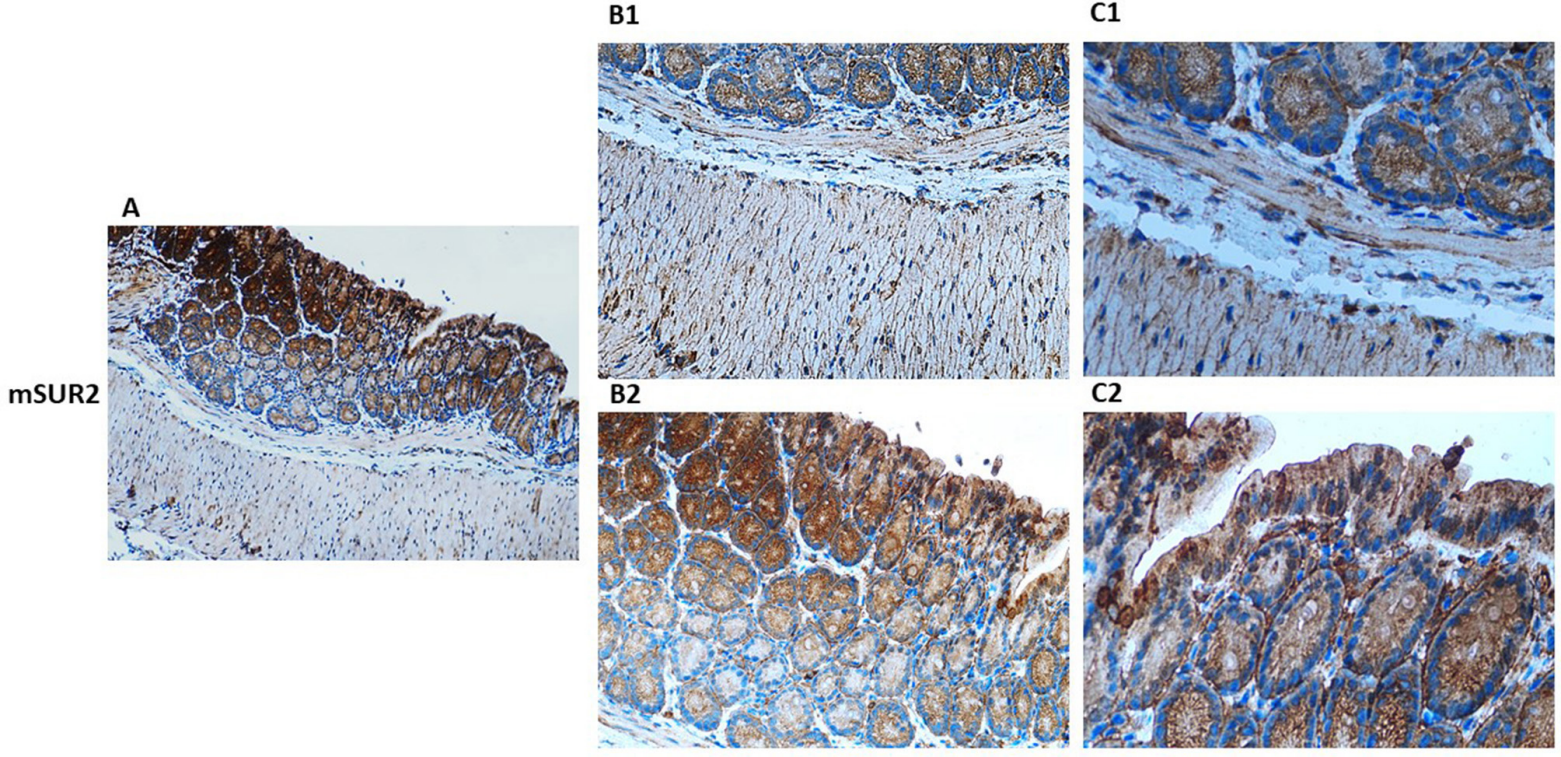
Immunohistochemical localization of KATP channel SUR2 subunit in normal mouse colon tissue. Representative immunohistochemical staining illustrating the distribution of KATP channel SUR2 subunit in histological sections of normal mouse colon at varying magnifications. **(A)** shows low-magnification imaging (10×) to provide an overview of overall tissue morphology. **(B1, B2)** (20×) and **(C1, C2)** (40×) present higher magnification views to resolve specific regional expression. At 20× and 40× magnification, Kir6.1 immunoreactivity is observed in distinct layers of the colon, including the lamina propria and muscularis **(B1, C1)**, as well as within the crypt structures **(B2, C2)**.

Histological analyses of human and murine colonic tissue reveal distinct localization of Kir6.1 and SUR2-gated KATP channels across epithelial and muscular compartments. Immunohistochemical staining demonstrates robust expression of Kir6.1 and SUR2 subunits within the smooth muscle layers, with pronounced enrichment in circular and longitudinal muscle fibers (Figures 1–3). These subunits are predominantly localized along the plasma membrane of smooth muscle cells, consistent with their role in modulating colonic motility through regulation of muscle tone and contractility in response to ATP and ADP fluctuations. Notably, Kir6.1 and SUR2 expression is also evident in ICC, which are distributed among smooth muscle fibers and concentrated within the myenteric and submucosal plexuses, suggesting their involvement in coordinating colonic pacemaker activity (34–36).

Within the epithelial compartment, Kir6.1 and SUR2 immunoreactivity is predominantly localized to the apical and basolateral membranes of enterocytes, with pronounced staining in the crypts of Lieberkühn (Figure 3). This distribution suggests a functional role in epithelial ion transport, particularly in chloride and water secretion, contributing to maintaining intestinal fluid homeostasis. Co-localization studies with Occludin and ZO-1 reveal the presence of Kir6.1 and SUR2 within tight junction complexes, implicating them in regulating epithelial barrier integrity. High-resolution imaging further demonstrates punctate staining along lateral cell membranes, suggesting potential interactions between KATP channels and junctional proteins, which may modulate paracellular permeability (20, 38).

Beyond epithelial and muscular compartments, Kir6.1 and SUR2 expression extends to neuronal structures of the enteric nervous system, with detectable levels in myenteric and submucosal ganglia. Within these regions, expression is particularly associated with inhibitory motor neurons, where KATP channel activation is linked to reduced excitability and modulation of enteric reflexes. This expression pattern aligns with electrophysiological studies implicating KATP channels in enteric neurotransmission (20). The spatially distinct distribution of Kir6.1 and SUR2 subunits across epithelium, smooth muscle, and neural plexuses underscores their multifaceted roles in colonic physiology, influencing both epithelial transport processes and neuromuscular regulation of peristalsis (Figures 1–3).

### GOF CS mutations result in an alteration in the mRNA expression of tight junction proteins (Occludin, Claudin-1, and ZO-1) in colon tissue

**Figure 4:**
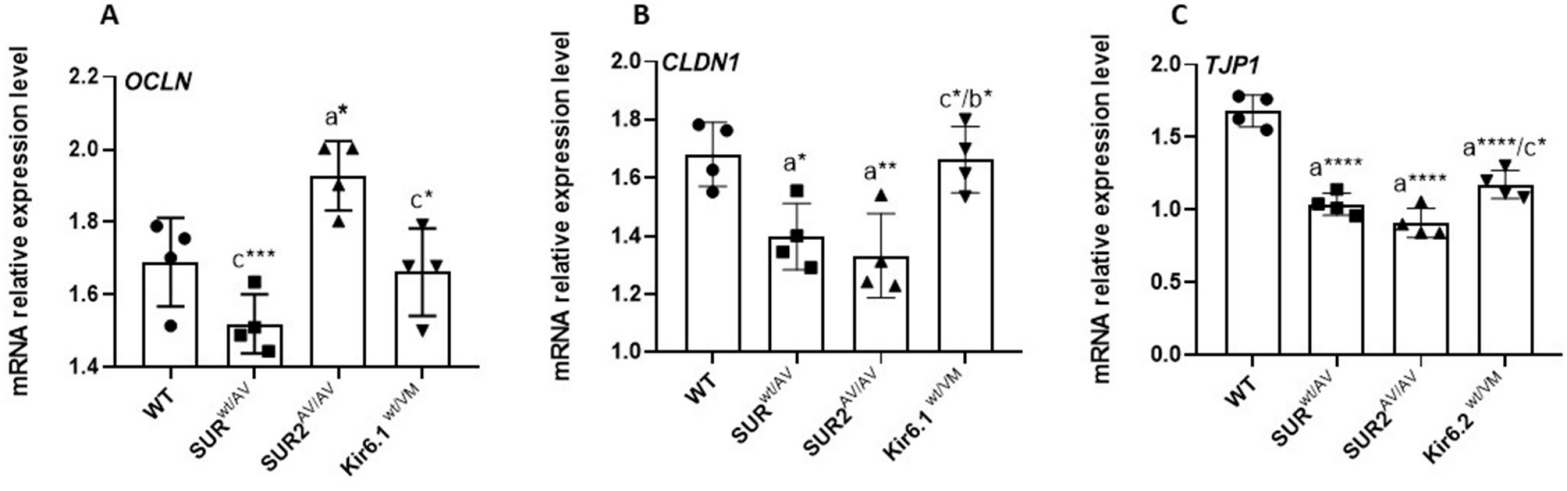
Quantitative RT-PCR analysis of tight junction gene expression. mRNA levels of *OCLN* **(A)**, *CLDN1* **(B)**, and *TJP1* [ZO-1; **(C)**] in colon tissue from WT and CS mice (SUR2^wt/AV^, SUR2^AV/AV^, Kir6.1^wt/VM^). Results are expressed as mean ± SD of a minimum of three independent experiments with three replicates per experimental condition in each experiment (n. animals used for these evaluations: 4 animals per genotype). a: significance vs. WT, b: significance vs. SUR2^wt/AV^, c: significance vs. SUR2^AV/AV^, and d: significance (p < 0.05) vs. Kir6.1wt/VM. One-way ANOVA followed by Tukey's *post hoc* test was used to evaluate statistical significance; *P < 0.05, **P < 0.01, ***P < 0.001, ****P < 0.0001.

Quantitative real-time PCR (qRT-PCR) analysis of colonic RNA extracted from CS and WT mice revealed significant changes in the mRNA expression profiles of key tight junction proteins, including Occludin, Claudin-1, and ZO-1, in mutant mice compared to controls. Specifically, the expression of Occludin, a critical tight junction protein responsible for regulating epithelial barrier integrity, was differentially regulated depending on the specific KATP mutation; Occludin expression was significantly (*p* < 0.05) upregulated in SUR2^AV/AV^ mice compared to WT controls. In contrast, substantial downregulation of Occludin was observed in SUR2^wt/AV^ and Kir6.1^wt/VM^ mice compared to SUR2^AV/AV^ mice, suggesting disruption in tight junction assembly and function. These alterations in Occludin expression may indicate an underlying increase in epithelial permeability, potentially contributing to the mutants' altered intestinal barrier function. Similarly, Claudin-1, another integral component of tight junctions known to play a role in maintaining the paracellular permeability barrier, showed a significant decrease in expression in SUR2^AV/AV^ and SUR2^wt/AV^ mice compared to WT mice. However, in Kir6.1^wt/VM^ mice, Claudin-1 expression was significantly increased compared to SUR2^AV/AV^, suggesting potential compensatory mechanisms or differential regulation in this specific genetic background. These changes indicate potential defects in establishing tight junctions that could compromise epithelial barrier function in these mutant strains. Additionally, ZO-1, a critical scaffolding protein that links transmembrane tight junction proteins to the actin cytoskeleton, showed a significant (p < 0.05) decrease in mRNA expression in SUR2^AV/AV^, SUR2^wt/AV^, and Kir6.1^wt/VM^ mice compared to WT controls. This reduction in ZO-1 transcript levels is consistent with structural and functional disruptions of tight junctions, which may further compromise the intestinal epithelium's integrity. These findings suggest a complex and multifactorial disruption of tight junction protein expression and assembly in mutant mice, potentially contributing to impaired epithelial permeability and barrier function. Results are expressed as mean ± SD (*n* = 3 biological replicates per group, each with three technical replicates). Statistical significance was assessed by one-way ANOVA with Tukey's *post hoc* test; exact *p*-values are reported in Figure 4.

### Gain-of-function (GOF) mutations in the CS gene enhance transcriptional activation of the intrinsic apoptotic machinery in the colon

**Figure 5:**
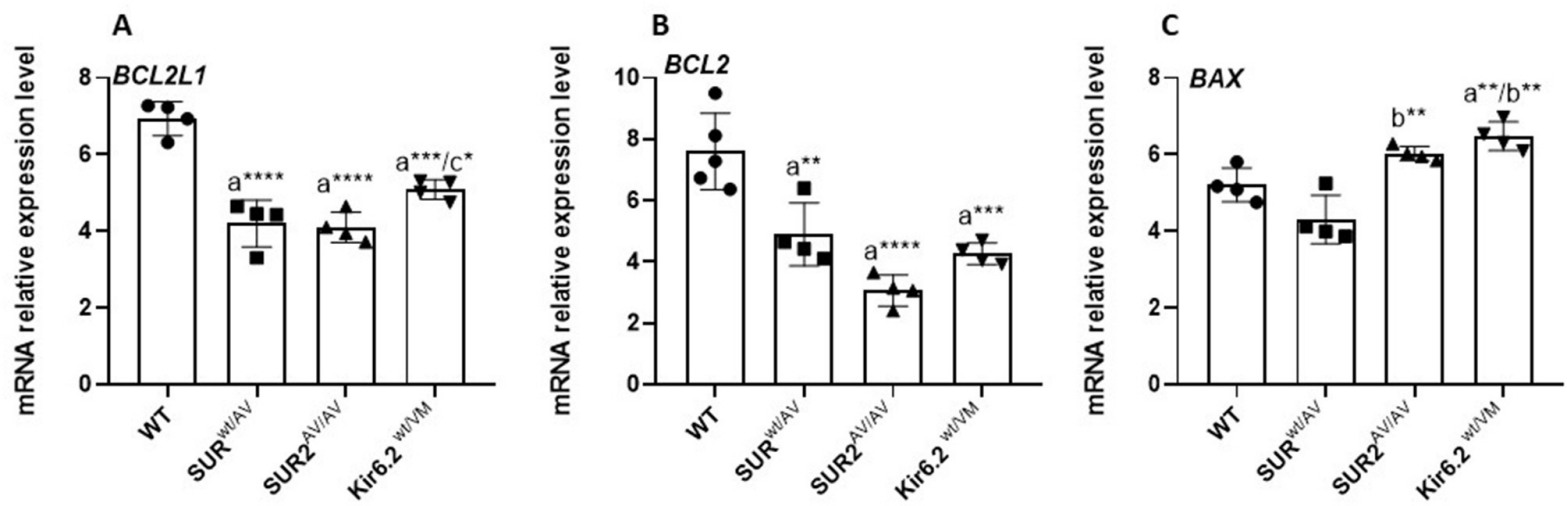
mRNA expression levels of *BCL2L1* **(A)**, *BCL2* **(B)**, and *BAX* **(C)** in colon tissue of CS mice vs. WT mice. Results are expressed as mean ± SD of a minimum of three independent experiments with three replicates per experimental condition in each experiment (n. animals used for these evaluations: 4 animals per genotype). a: significance vs. WT, b: significance vs. SUR2wt/AV, c: significance vs. SUR2AV/AV, and d: significance (*p* < 0.05) vs. Kir6.1wt/VM. One-way ANOVA followed by Tukey's *post hoc* test was used to evaluate statistical significance; *P < 0.05, **P < 0.01, ***P < 0.001.

Gain-of-function (GOF) mutations in the CS gene selectively modulate the transcriptional profile of apoptotic regulators in the colon. RT–qPCR analysis revealed a significant (*p* < 0.05) downregulation of anti-apoptotic *BCL2L2* and *BCL2* mRNA levels in colonic tissue from SUR2^wt/AV^, SUR2^AV/AV^, and Kir6.1^wt/VM^ mice compared to wild-type controls. In contrast, the pro-apoptotic gene *BAX* expression remained unchanged (*p* > 0.05) across all CS mutant models. This selective repression of anti-apoptotic transcripts, without a compensatory increase in pro-apoptotic signaling, suggests a disrupted apoptotic equilibrium that may underlie aberrant epithelial turnover and prolonged cell survival. These findings point to a previously unrecognized impact of CS GOF mutations on apoptotic homeostasis in the colon, with potential implications for tissue integrity and disease susceptibility (Figure 5).

**Figure 6:**
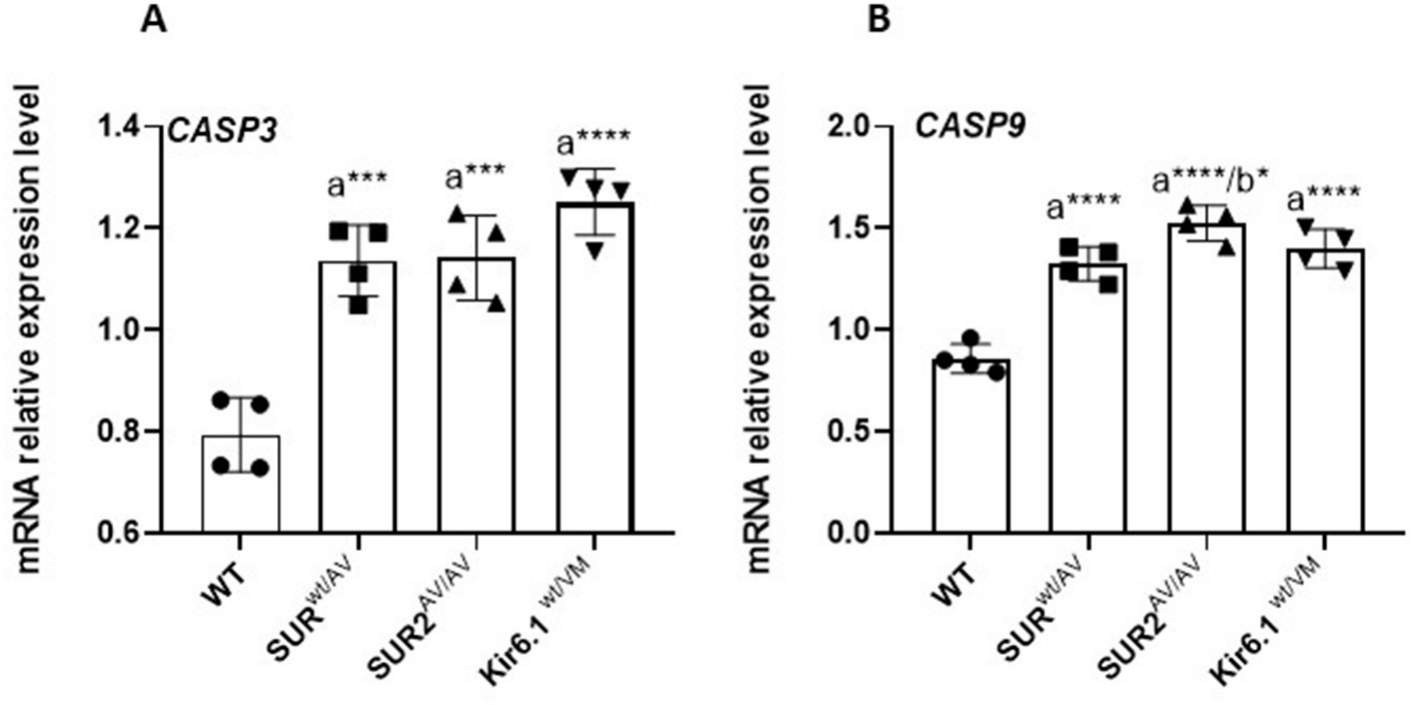
mRNA expression levels of *CASP3* **(A)** and *CASP9* **(B)** in CS mice and WT mice's colon tissue. Results are expressed as mean ± SD of a minimum of three independent experiments with three replicates per experimental condition in each experiment (n. animals used for these evaluations: 4 animals per genotype). a: significance vs. WT, b: significance vs. SUR2wt/AV, c: significance vs. SUR2AV/AV, and d: significance (*p* < 0.05) vs. Kir6.1wt/VM. One-way ANOVA followed by Tukey's *post hoc* test was used to evaluate statistical significance; *P < 0.05, **P < 0.01, ***P < 0.001.

Gain-of-function mutations in the CS gene result in a transcriptional shift toward pro-apoptotic signaling in the colon, a process closely intertwined with oxidative stress responses. In colon tissue from SUR2^wt/AV^, SUR2A^V/AV^, and Kir6.1^wt/VM^ mice, RT–qPCR analysis revealed significant upregulation of *CASP9* and *CASP3*, central mediators of the mitochondrial (intrinsic) apoptotic pathway, along with reduced expression of the anti-apoptotic genes *BCL2* and *BCL2L2* (Bcl-xL). This molecular profile is consistent with increased susceptibility to mitochondrial outer membrane permeabilization and caspase activation in response to cellular stress (Figure 6).

### Gain-of-function CS mutations lead to the disruption of tight junction protein expression and epithelial barrier integrity

**Figure 7:**
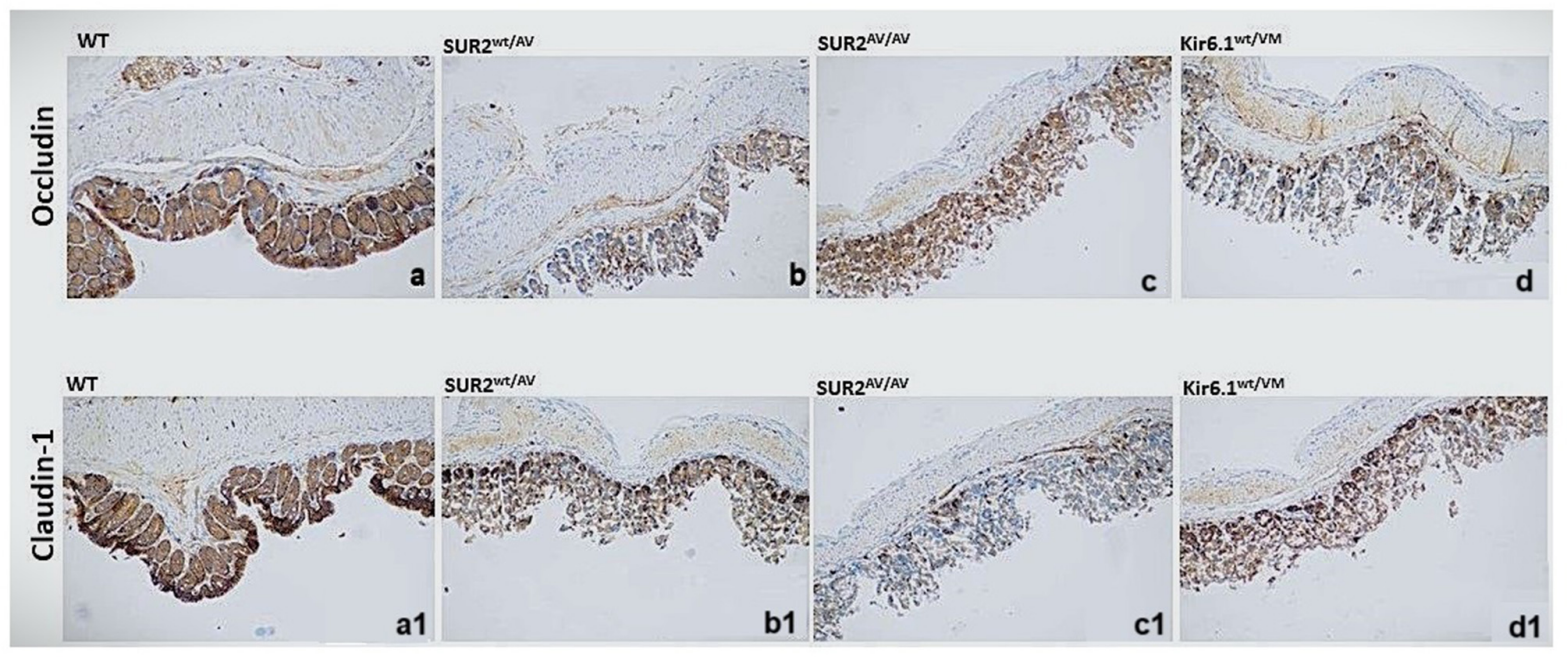
Representative histological analysis of the sample with hematoxylin-eosin (H&E) reaction and immunostaining for junction proteins (occludin, and Claudin-1) on colon sections of **(a, a1)** WT, **(b, b1)** SUR2^wt/AV^ **(c, c1)** SUR2^AV/AV^ and **(d, d1)** Kir6.1^wt/VM^ mice at 20×. (n. animals used for these evaluations: 4 animals per genotype.

Immunohistochemical staining of colonic sections from CS and WT mice revealed significant alterations in the expression of tight junction proteins, particularly Occludin and Claudin-1. In WT mice, both proteins were strongly expressed at the apical junctions of epithelial cells, maintaining the characteristic organization of tight junctions. In contrast, CS mice exhibited a marked reduction in the expression of Occludin and Claudin-1, particularly at intercellular junctions. The staining intensity of Occludin was notably diminished, indicating a disruption in tight junction integrity, while Claudin-1 expression was similarly reduced, further suggesting impaired barrier function. This decrease in tight junction protein expression may underlie a structural and functional impairment of the epithelial barrier, potentially contributing to increased intestinal permeability in CS mice. These findings support the hypothesis that altered KATP channel activity plays a role in destabilizing tight junctions, which may exacerbate barrier dysfunction and intestinal inflammation in the mutant phenotype (Figure 7).

### Gain-of-function CS mutations induce structural and functional alterations in the colonic epithelium and smooth muscle of CS mice

**Figure 8:**
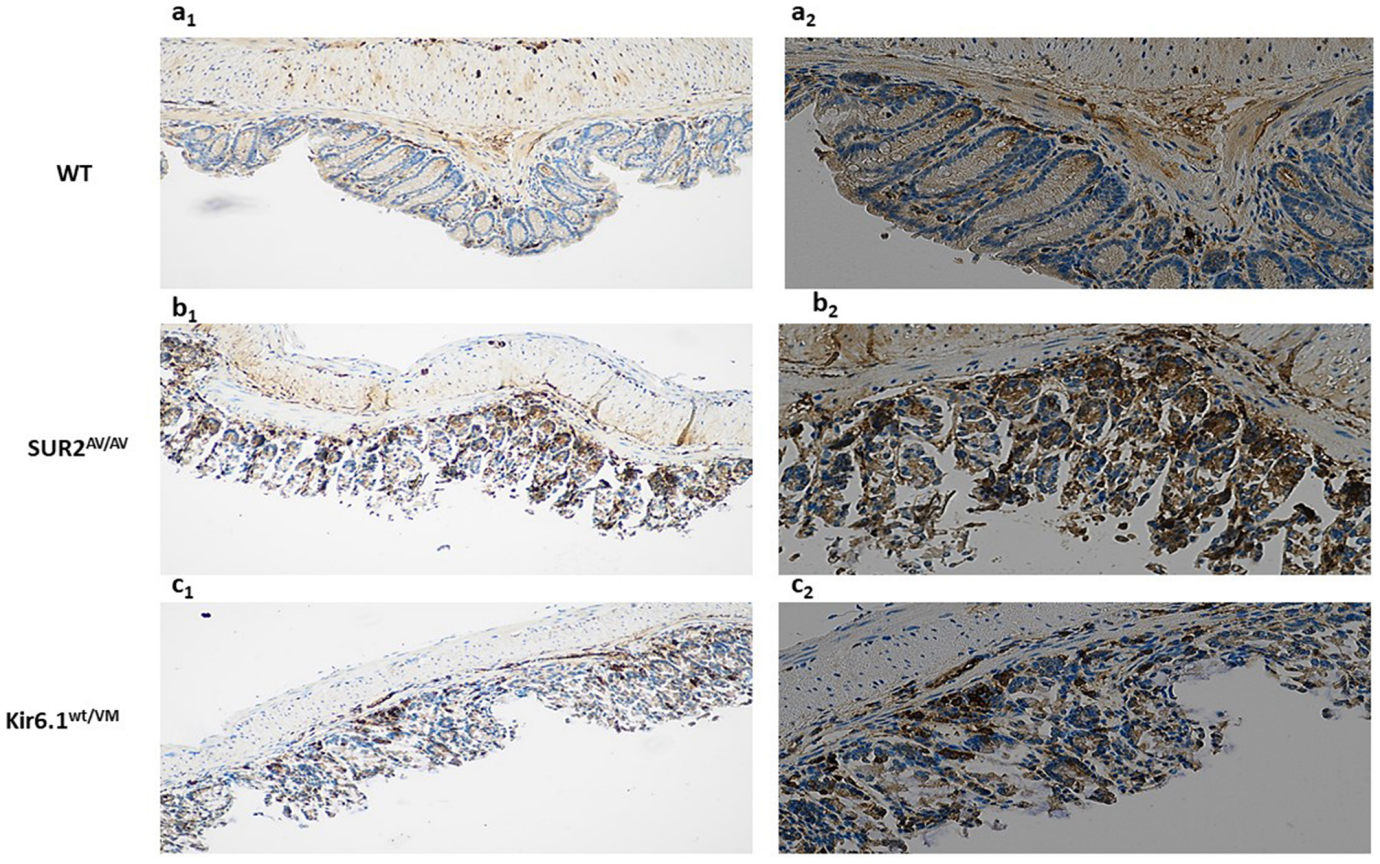
Representative histological analysis of the sample with hematoxylin-eosin (H&E) reaction and immunostain for casp-3 on colon sections of **(a**_**1**_**, a**_**2**_**)** WT, **(b**_**1**_**, b**_**2**_**)** SUR2^AV/AV^ SUR2^wt/AV^ and **(c**_**1**_**, c**_**2**_**)** Kir6.1^wt/VM^ mice at 20× (left) and 40× (right; n. animal used for these evaluations: 3 animals per genotype).

Histological examination of colonic sections from CS and WT mice revealed notable structural alterations in the colonic epithelial layer and smooth muscle of CS mice. CS mice disrupted the typical organization of tightly packed columnar cells with well-defined apical-basal polarity in the epithelial layer. The epithelium exhibited signs of disorganization, including irregular cell arrangement and a marked increase in intercellular spaces, indicative of a compromised epithelial barrier. This disruption is likely associated with the destabilization of tight junctions, as evidenced by the reduced expression of tight junction proteins, such as Occludin and Claudin-1. Moreover, the epithelial layer of CS mice showed increased cellular infiltration, indicative of an inflammatory response, which further supports the notion of epithelial dysfunction. Notably, sections from CS mice exhibited autolytic disintegration phenomena, likely a consequence of the inflammatory state, accompanied by intense caspase-3 immunoreactivity (Figure 8).

CS mice displayed hypertrophic changes in the underlying smooth muscle layer characterized by increased muscle thickness and apparent disorganization of smooth muscle fibers. Smooth muscle cells appeared structurally disordered and showed signs of hyperplasia, particularly in the muscularis propria, most prominently in SUR^AV/AV^ mice. These structural changes were accompanied by increased fibrosis and heightened extracellular matrix deposition. This fibrotic remodeling suggests potential smooth muscle dysfunction, possibly contributing to impaired motility and contractility in CS mice (Table 1).

**Table 1:** A comparative histological analysis was performed to assess the severity and distribution of pathological changes in colonic tissue across genotypes, including WT and CS mice (SUR2^wt/AV^, SUR2^AV/AV^, Kir6.1^wt/VM^).

**Pathological changes**	**WT**	**SUR2^wt/AV^**	**SUR2^AV/AV^**	**Kir6.1^wt/VM^**
Diffuse inflammation	–/+	++	+++	+++
Patchy/focal inflammation	–	++	+++	+
Active cryptitis	–	+++	+++	+++
Mucosal remodeling	–	+++	+++	+++
Alteration of the musculature	–	++	++	+++
Neutrophils in lamina propria	–	++	++	++

Overall, these structural alterations in the epithelial and smooth muscle layers of CS mice reflect a complex disruption of colonic architecture, likely contributing to observed physiological abnormalities such as increased intestinal permeability and impaired motility. These impairments may stem from altered KATP channel activity in CS mice, which exacerbates both epithelial and smooth muscle dysfunction, ultimately contributing to the pathophysiology of the mutant phenotype (Figure 8, Table 1). These alterations reflect a shared pathological signature similar to chronic inflammatory bowel disease, with the most severe manifestations in SUR2^AV/AV^ and Kir6.1^wt/VM^ mice.

## Discussion

To our knowledge, this study provides the first comprehensive evidence that Kir6.1 and SUR2 subunits of KATP channels are strongly expressed in both human and murine colons, with predominant localization in the colonic epithelium. These findings suggest that KATP channels are crucial in maintaining intestinal barrier integrity, enabling selective nutrient absorption while preventing pathogen infiltration. Prior research has documented the presence of Kir6.1/SUR2 complexes in rodents' non-vascular smooth muscle and colonic epithelial cells, and their co-localization with TJ proteins in the small intestine (40, 43). Altogether, these data highlight the multifaceted role of KATP channels in epithelial and endothelial homeostasis.

KATP channels are well-known metabolic sensors that couple ATP/ADP ratios to ion fluxes and membrane potential, ensuring cellular adaptation to metabolic stressors such as hypoxia or ischemia (13, 16). They regulate apical ion transport in the colon, particularly chloride secretion, as shown in rodent models (26, 45).

We employed multiple murine models carrying gain-of-function (GOF) mutations in SUR2 [SUR2(A478V)] or Kir6.1 [Kir6.1(V65M)] to explore the impact of KATP channel hyperactivity. Heterozygous SUR2A478V animals showed moderate epithelial alterations, while homozygous mutants displayed more severe pathology, including smooth muscle hypertrophy, fibrosis, and inflammation, consistent with a dose-dependent GOF effect. Kir6.1V65M mice exhibited pronounced deficits in motility and neuromuscular coordination, which aligns with findings by York et al. (20), who demonstrated that GOF mutations impair gastrointestinal contractility. Pharmacological activation with pinacidil inhibited motility, whereas glibenclamide reversed transit deficits, especially in Kir6.1^wt/VM^ mice.

In WT mice, epithelial homeostasis is maintained through cell turnover, TJ remodeling, and immune surveillance. In contrast, CS mice with GOF mutations in Kir6.1 or SUR2 displayed compromised barrier function and heightened inflammation (20). Histological and RT-qPCR analyses revealed that WT colons had normal expression of TJ proteins such as Occludin and Claudin-1, essential for controlling paracellular permeability. CS mice, however, exhibited significantly reduced expression of these proteins, confirmed by diminished immunoreactivity in tissue sections hallmarks of impaired barrier function. So, Occludin levels are differentially regulated depending on the specific KATP mutation: upregulated in *SUR*2^*A*^*V*/*AV* mice and downregulated in *SUR*2^*w*^*t*/*AV* and *Kir*6.1^*V*^65*M* mutants. These differential expression patterns may reflect mutation-specific effects on tight junction dynamics and compensatory responses, rather than uniform dysregulation.

Our data suggest that KATP channel hyperactivity compromises TJ integrity through converging mechanisms, notably membrane hyperpolarization and overproduction of reactive oxygen species (ROS) (29, 30). TJs are composed of transmembrane proteins (e.g., claudins, occludins), cytoplasmic scaffolding proteins (e.g., ZO-1, ZO-2, ZO-3), and actin cytoskeleton components (46–48). ROS-induced actin depolymerization can destabilize the junctional complex, increasing permeability (47, 49). Electrolyte imbalance and metabolic dysregulation in CS mice likely exacerbate TJ disruption.

GOF mutations in KATP subunits led to structural and molecular abnormalities, including epithelial disorganization, inflammation, and apoptosis. These defects were accompanied by increased caspase-3 activity, indicating widespread programmed cell death. Mechanistically, sustained potassium efflux and plasma membrane hyperpolarization elevate intracellular calcium, enhancing mitochondrial respiration and generating excess ROS. This oxidative stress damages TJ components, disrupts cytoskeletal anchoring, and activates inflammatory pathways. It also perturbs apoptotic regulation by decreasing *BCL2/BCL-XL* and upregulating *BAX*, further driving caspase-3-mediated cell death and tissue disintegration.

Additionally, KATP channels may directly interact with TJ components, as shown by the co-localization of Kir6.1 and SUR2 with Claudin-1, implicating a structural role in barrier maintenance.

Beyond epithelial cells, our localization studies revealed expression of Kir6.1 and SUR2 in colonic smooth muscle and ICC, suggesting involvement in neuromuscular coordination and pacemaker activity. This may underlie the motility impairments seen in CS models, especially in Kir6.1 mutant.

Overall, our findings point to KATP channel dysregulation as a central driver of intestinal dysfunction in CS via oxidative stress, inflammatory activation, and epithelial disruption. However, the precise molecular pathways, particularly calcium signaling and ROS, warrant further investigation.

This study provides novel insights into the role of KATP channels in CS-related intestinal pathology. Its strength lies in using multiple murine models and a multi-level analytical approach encompassing molecular, histological, and functional parameters. Identifying oxidative stress and apoptosis as mediators of TJ dysfunction highlights promising therapeutic targets.

Nevertheless, some limitations should be acknowledged. While murine models offer valuable mechanistic insights, interspecies differences in physiology and immune responses may limit translation to humans. Our analyses focused primarily on structural, molecular and histological features of colonic epithelial integrity. Functional permeability assays, such as Using chamber measurements or sugar absorption tests, were not performed, representing an important limitation. Such assays provide direct quantification of paracellular flux, electrolyte transport and barrier function, and would be essential to corroborate the molecular observations derived from frozen tissues, which are well suited for immunohistochemistry and histochemical analyses but not for functional studies. By concentrating on the colon, our work does not address potential contributions from other intestinal regions. Although the colon was selected owing to its established relevance in disease, the small intestine differs substantially in immune cell populations, microbial ecology, nutrient absorption and epithelial turnover, all of which may shape pathophysiology and responses to intervention. Further, direct assessments of intracellular calcium flux and reactive oxygen species generation would strengthen the mechanistic link between KATP channel hyperactivity, oxidative stress and tight junction disruption. Future studies will therefore aim to define the causal chain connecting KATP hyperactivity, calcium flux and ROS production, incorporating Ussing chamber assays and complementary measures of epithelial transport to validate the structural findings reported here. Finally, although CS is not generally associated with sex differences in cardiovascular function, possible sex-specific effects of CS mutations on intestinal physiology cannot be excluded, and forthcoming experiments in CS mice will address this question.

Despite these limitations, this work lays the foundation for future research to develop therapeutic strategies targeting KATP channels or downstream pathways to restore intestinal barrier function in CS and related conditions.

## Materials and methods

### Animal care and ethical statements

Given the current lack of evidence supporting sex-based differences in conditioned stimulus (CS) responses, our experiments were conducted exclusively using male mice to maintain consistency and reduce potential variability (8, 9). Despite this, we are aware that sex differences may influence epithelial and immune function, potentially affecting disease pathophysiology and therapeutic responses due to hormonal influences. Male knock-in mice carrying Kir6.1(V65M) (Kir6.1^*w*^*t*/*VM*), SUR2(A478V) heterozygous (SUR2^*w*^*t*/*AV*), and SUR2(A478V) homozygous (SUR2^*A*^*V*/*AV*) mutations mimicking human Cantú syndrome (CS) were generated via CRISPR/Cas9 gene editing at Washington University in St. Louis, USA, and subsequently transferred to Italy. Genotyping was performed as described by Huang et al. (8, 9). Animals were housed in groups of two to four per cage at the Animal Facility of the Department of Pharmacy-Drug Sciences, University of Bari, Italy. Housing conditions were maintained at 22 ± 1 °C with 50 ± 5% relative humidity and a 12:12 light/dark cycle, with *ad libitum* access to a standard laboratory diet and water. All experimental procedures adhered to European Directive 2010/63/EU on the protection of animals used for scientific purposes and were approved by the Institutional Animal Care and Use Committee (IACUC) of Washington University School of Medicine, as well as by the Italian Ministry of Health and the University of Bari's OPBA (Organization for Animal Health; protocol 8515-X/10, January 30, 2019).

### Animal sacrifice and tissue harvesting

Animals were sacrificed by cervical dislocation under deep anesthesia with ZOLETIL 50/50 (40 mg/kg, i.p.). Tissues and organs were harvested under sterile conditions to prevent contamination. Organs were weighed and either snap-frozen in liquid nitrogen for protein extraction or embedded in OCT for immunofluorescence and immunohistochemistry, then stored at −80 °C. All procedures were conducted in Ringer's solution (145 mM NaCl, 5 mM KCl, 1 mM MgCl_2_, 0.5 mM CaCl_2_, 5 mM glucose, 10 mM MOPS, pH 7.2).

### Total RNA isolation and RT-qPCR

According to the manufacturer's instructions, total RNA was isolated from colon tissue using TRIzol reagent (Invitrogen, Waltham, MA, USA). RNA purity and concentration were assessed with a NanoDrop ND-1000 spectrophotometer (Thermo Fisher Scientific, Waltham, MA, USA), while RNA integrity was confirmed by electrophoresis on a denaturing agarose gel. Complementary DNA (cDNA) was synthesized via reverse transcription. Gene expression levels were quantified by real-time quantitative PCR (RT-qPCR) using a 2 × SYBR Green master mix (Applied Biosystems, Foster City, CA, USA) following the manufacturer's guidelines. Primer sequences for RT-qPCR are listed in Table 2. Relative gene expression was determined by the ΔCT method, with *GAPDH* as the internal control for normalization.

**Table 2:** Primers used in RT-qPCR.

**Gene**	**Forward**	**Reverse**
*OCLN*	CTCCCATCCGAGTTTCAGGT	GCTGTCGCCTAAGGAAAGAG
*CLDN1*	GTTTGCAGAGACCCCATCAC	AGAAGCCAGGATGAAACCCA
*TJP1*	CTGAGTTGCCCGCGACG	CTGCTCCCGCACGTAACTTC
*CASP9*	AGTTCCCGGGTGCTGTCTAT	GCCATGGTCTTTCTGCTCAC
*CASP3*	CCTCAGAGAGACATTCATGG	GCAGTAGTCGCCTCTGAAGA
*BCL2*	CTCGTCGCTACCGTCGTGACTTCG	CAGATGCCGGTTCAGGTACTCAGTC
*BCL2L1*	TGGAGTAAACTGGGGGTCGCATCG	AGCCACCGTCATGCCCGTCAGG
*BAX*	AAGCTGAGCGAGTGTCTCCGGCG	GCCACAAAGATGGTCACTGTCTGCC
*GAPDH*	GCCCAATACGACCAAATCC	AGCCACATCGCTCAGACAC

### Immunohistochemical and histological analysis

Formalin-fixed, paraffin-embedded (FFPE) tissue specimens were sectioned at 4 μm, mounted on Apex Bond Slides (Leica Biosystems), and subjected to immunohistochemical and histological analyses. Immunohistochemistry was performed using a BOND III automated stainer (Leica Biosystems, Wetzlar, Germany) with the Bond Polymer Refine Detection Kit, encompassing deparaffinization through hematoxylin counterstaining. Sections were incubated for 30 min at room temperature with primary antibodies against Kir6.1 (monoclonal, AB174629, Abcam, Toronto, Canada), SUR2 (monoclonal, AB271996; Abcam, Toronto, Canada), Caspase-3 (polyclonal, 9662; Cell Signaling Technology, Danvers, MA, USA), Occludin (monoclonal, 91131; Cell Signaling Technology), and Claudin-1 (polyclonal, 4933; Cell Signaling Technology). Antigen retrieval was performed using BOND Epitope Retrieval Solution 2 (Leica Biosystems). For histological evaluation, sections were stained with hematoxylin and eosin (H&E) and reviewed by a board-certified pathologist. Inflammatory scoring was based on epithelial integrity, immune cell infiltration, and tissue/muscle remodeling, whereas morphological assessment included crypt architecture, goblet cell preservation, and mucosal organization. Pathological alterations were quantified using standardized criteria to ensure reproducibility. Images were captured using a Nikon Eclipse Ti2 microscope.

### Data analysis and statistics

All data were collected and analyzed using Excel (Microsoft Office 2010) and SigmaPlot 10.0 (Systat Software). Results are presented as mean ± SEM unless otherwise specified. The corresponding figure legends detail the number of biological and technical replicates for each experiment. Statistical analyses were conducted using one-way analysis of variance (ANOVA) followed by *post hoc* multiple comparisons to assess differences among groups, with a significance level of *p* < 0.05 unless otherwise stated. Student's *t*-test was applied for pairwise comparisons, with statistical significance defined as *p* < 0.05. Sample sizes were determined via power analysis (α = 0.05, power = 0.8) based on pilot data. Experimental groups included Kir6.1^wt/VM^ (*n* = 4), SUR2^wt/AV^ (*n* = 4), SUR2^AV/AV^ (*n* = 4), and wild-type (WT) controls (*n* = 4). These sample sizes were chosen based on previous studies in similar models, which consistently demonstrated robust and reproducible effects using comparable cohorts, particularly for mechanistic and histological endpoints. Investigators were blinded to genotype during data collection and analysis. Lower power of 0.72 is calculated with a sample size of *n* = 3.

## Conclusion

The present study demonstrates that GOF mutations in Kir6.1 and SUR2, characteristic of CS, induce profound alterations in TJ protein expression and epithelial barrier integrity. CS murine models exhibited disrupted mRNA and protein levels of Occludin, Claudin-1, and ZO-1, correlating with structural epithelial defects, increased paracellular permeability, and inflammatory infiltration. Additionally, smooth muscle hypertrophy and fibrosis indicate broader neuromuscular dysfunction in the colon.

Mechanistically, KATP channel hyperactivity likely drives oxidative stress and caspase-3-mediated apoptosis, compromising barrier integrity and sustaining a cycle of inflammation and tissue damage. These findings underscore the essential role of KATP channels in intestinal homeostasis and link their dysregulation to epithelial and neuromuscular pathology in CS.

Given that SUR1 and SUR2 subunits are well-established drug targets in diabetes and cardiovascular diseases, the high expression of KATP channel subunits in the colon suggests their potential for pharmacological modulation of intestinal motility and barrier function. Targeting oxidative stress or KATP channel activity may offer novel therapeutic strategies to restore barrier function and mitigate intestinal complications in CS and related disorders.

## Data Availability

The original contributions presented in the study are included in the article/supplementary material, further inquiries can be directed to the corresponding author.
